# Identification of oral symptoms associated with atopic dermatitis in adolescents: Results from the Korea national representative survey 2009–2017

**DOI:** 10.1038/s41598-020-76532-1

**Published:** 2020-11-10

**Authors:** Ji-Su Shim, Min-Suk Yang

**Affiliations:** 1 Division of Allergy and Clinical Immunology, Department of Internal Medicine, Ewha Womans University College of Medicine, Seoul, Korea; 2 Division of Allergy and Clinical Immunology, Department of Internal Medicine, Seoul National University College of Medicine, Seoul, Korea; 3 Department of Internal Medicine, SMG-SNU Boramae Medical Center, 5 Gil 20 Boramae-Road, Dongjak-gu, Seoul, 07061 Korea

**Keywords:** Oral manifestations, Skin diseases, Risk factors

## Abstract

The relationship between oral health and atopic dermatitis (AD) remains unclear. Here we investigated the association between oral health status and AD using data from 634,299 subjects in the Korean Youth Risk Behavior Survey (KYRBS). Participants with oral symptoms were defined as those with any of following: sensitive teeth, toothache, bleeding gums or gum pain, and dry mouth. Current AD was determined by the question if participant had been diagnosed with AD from doctor within the past 12 months. We estimated the odds ratio (OR) for AD diagnosis according to the presence of oral symptoms. The OR for current AD, which is a dependent variable, was significantly increased in participants with oral symptoms, which are independent variables, in an adjusted model (OR, 1.27; 95% confidence interval [CI], 1.26–1.29; *P* < 0.001). In detailed analyses, all four oral symptoms were significantly associated with AD diagnosis: sensitive teeth (OR, 1.21; CI, 1.19–1.23; *P* < 0.001), bad breath (OR, 1.18; CI, 1.17–1.20; *P* < 0.001), toothache (OR, 1.18; CI, 1.16–1.20; *P* < 0.001), and bleeding gums (OR, 1.14; CI, 1.12–1.16; *P* < 0.001). In the presence of oral symptoms, the ORs for having two or more allergic diseases (AD, allergic rhinitis, and/or asthma) were higher than that of AD alone. In this study, oral symptoms appeared to be associated with AD in Korean adolescences.

## Introduction

Atopic dermatitis (AD), a common allergic disease during childhood, is a chronic and relapsing inflammatory skin disease, often preceded by allergic rhinitis (AR) and asthma^1^. The prevalence of AD has been reported as 10–30% in children and 2–17% in adults^2–4^, and it has been increasing over the last 30 years not only in western countries, such as Western Europe and the United States, but also in Africa, Northern Europe, the Middle East, and East Asia^5,6^. In Korea, 13.5% of children and adolescents have AD^7^.

AD, AR, and asthma are typical “atopic” diseases, and are commonly associated with T helper type 2 (Th2) inflammatory pathway, which characterized by production of allergen-specific immunoglobulin E and Th2-type cytokines such as interleukin (IL)-4, IL-5, and IL-13, and eosinophilic infiltration^8,9^. Although a progression of allergic conditions in childhood, so called the “atopic march”, is well established^8^, there are still children and adolescents with a single allergic disease only. The mechanism of this susceptibility difference is not known exactly, but may arise from the differences in structural, genetic, environmental, and immunological factors. In case of AD, skin barrier dysfunction is considered as an important pathogenesis.

The pathophysiology of AD is recognized as complex and multifactorial, and still under investigation^10^. Both skin barrier dysfunction and immune dysregulation have been implicated as major pathophysiological mechanisms underlying the development of AD, and current concepts suggest that defective skin barrier function is a primary process driving AD, not a consequence of the disease^10,11^. Although many genetic mutations regarding AD have been identified, which involve skin barrier function, environmental sensing, innate and adaptive immune regulation, and tissue responses, the strongest genetic risk factor is a loss-of-function mutation in filaggrin (FLG), which plays an important role in maintenance of the skin barrier^1^. FLG aggregates keratin filaments in epithelial cells, and at least 20 mutations in FLG have been found in AD patients^1,10,12,13^.

A recent pediatric study found higher serum levels of FLG in patients with AR and asthma as well as AD, suggesting its potential pathological role in other atopic diseases not directly involving the skin^14^. In addition, immunohistochemical analyses have shown that FLG is expressed not only in the epidermis but also in the human oral mucosa^15^. Since disruption of barrier function could lead to frequent microbial infections and increased transepidermal water loss^11^, AD patients with structural skin abnormalities and disrupted integrity of the oral mucosa may have altered oral conditions, resulting in AD-associated oral manifestations that have yet to be documented fully. Since there was a large population-based national survey of adolescents in Korea, which included the questions on oral symptoms, such as sensitive teeth, toothache, bleeding or painful gums, and bad breath, we decided to investigate the association between the presence of AD and available oral symptoms in Korean adolescents using this data. Our hypothesis is that adolescents with oral symptoms may have a higher prevalence of AD.

## Results

### General characteristics

A total of 634,299 participants were included in this study, and the general characteristics of the total study population are presented in Table 1. The median age was 15.06 years, and the prevalence rates of physician-diagnosed current AD, AR, and asthma were 23.3%, 32.9%, and 9.0%, respectively. Of all participants, 59.8% reported that they had experienced oral symptoms. The detailed general characteristics according to the presence of allergic disease such as current AD, AR, and asthma are also presented in Table 1.

**Table 1:** General characteristics of the total study population.

Characteristic/s	Total	Atopic dermatitis		P-value	Allergic rhinitis		P-value	Asthma		P-value
		No	Yes		No	Yes		No	Yes	
**Total**										
n	634,299	487,520	146,779		431,446	202,853		577,857	56,442	
%	100.0	76.7 ± 0.1	23.3 ± 0.1		67.1 ± 0.1	32.9 ± 0.1		91.0 ± 0.0	9.0 ± 0.0	
**Age (year)**	15.1 ± 0.0	15.1 ± 0.0	15.0 ± 0.0	< 0.001	15.0 ± 0.0	15.2 ± 0.0	< 0.001	15.1 ± 0.0	15.0 ± 0.0	< 0.001
**Male (%)**	52.4 ± 0.5	54.5 ± 0.5	45.2 ± 0.5	< 0.001	52.5 ± 0.5	52.0 ± 0.5	< 0.001	51.8 ± 0.5	58.5 ± 0.5	< 0.001
**Region of residence (%)**				< 0.001			< 0.001			0.118
Large city	45.4 ± 0.6	45.4 ± 0.6	45.5 ± 0.6		45.4 ± 0.6	45.4 ± 0.7		45.5 ± 0.6	44.9 ± 0.7	
Medium or small city	48.4 ± 0.7	48.2 ± 0.7	48.8 ± 0.7		47.7 ± 0.7	49.6 ± 0.7		48.3 ± 0.7	49.4 ± 0.7	
Rural area	6.2 ± 0.2	6.4 ± 0.3	5.6 ± 0.2		6.8 ± 0.3	4.9 ± 0.2		6.3 ± 0.2	5.7 ± 0.3	
**Family income (%)**				< 0.001			< 0.001			0.014
Highest	7.7 ± 0.1	7.9 ± 0.1	7.0 ± 0.1		7.6 ± 0.1	7.8 ± 0.1		7.6 ± 0.1	8.7 ± 0.2	
Upper middle	25.2 ± 0.1	25.1 ± 0.1	25.5 ± 0.2		24.2 ± 0.1	27.4 ± 0.2		25.1 ± 0.1	26.0 ± 0.2	
Middle	47.1 ± 0.1	47.1 ± 0.1	47.1 ± 0.2		47.7 ± 0.1	45.9 ± 0.2		47.4 ± 0.1	44.3 ± 0.3	
Low middle	15.6 ± 0.1	15.5 ± 0.1	16.1 ± 0.1		16.0 ± 0.1	15.0 ± 0.1		15.6 ± 0.1	16.0 ± 0.2	
Lowest	4.4 ± 0.1	4.4 ± 0.1	4.2 ± 0.1		4.6 ± 0.1	3.9 ± 0.1		4.3 ± 0.0	5.0 ± 0.1	
**Smoking (%)**	9.8 ± 0.1	10.2 ± 0.1	8.5 ± 0.1	< 0.001	10.1 ± 0.1	9.2 ± 0.1	< 0.001	9.6 ± 0.1	11.2 ± 0.2	< 0.001
**Stress (%)**				< 0.001			< 0.001			< 0.001
Very high	11.1 ± 0.1	10.7 ± 0.1	12.3 ± 0.1		10.5 ± 0.1	12.4 ± 0.1		10.8 ± 0.1	13.7 ± 0.2	
High	29.0 ± 0.1	28.4 ± 0.1	31.3 ± 0.2		28.0 ± 0.1	31.1 ± 0.1		28.9 ± 0.1	30.6 ± 0.2	
Moderate	42.1 ± 0.1	42.4 ± 0.1	41.3 ± 0.2		42.6 ± 0.1	41.2 ± 0.1		42.3 ± 0.1	40.1 ± 0.2	
Low	14.8 ± 0.1	15.4 ± 0.1	13.0 ± 0.1		15.7 ± 0.1	13.1 ± 0.1		15.0 ± 0.1	13.0 ± 0.2	
None	2.9 ± 0.0	3.1 ± 0.0	2.2 ± 0.0		3.2 ± 0.0	2.3 ± 0.0		2.9 ± 0.0	2.7 ± 0.1	
**School performance (%)**				< 0.001			< 0.001			< 0.001
Highest	11.7 ± 0.1	11.5 ± 0.1	12.2 ± 0.1		10.7 ± 0.1	13.7 ± 0.1		11.6 ± 0.1	12.9 ± 0.2	
Middle high	24.5 ± 0.1	24.0 ± 0.1	26.0 ± 0.1		23.2 ± 0.1	27.1 ± 0.1		24.4 ± 0.1	25.3 ± 0.2	
Middle	27.7 ± 0.1	27.8 ± 0.1	27.3 ± 0.1		27.9 ± 0.1	27.2 ± 0.1		27.8 ± 0.1	26.4 ± 0.2	
Middle low	24.6 ± 0.1	24.8 ± 0.1	24.0 ± 0.1		25.7 ± 0.1	22.4 ± 0.1		24.7 ± 0.1	23.7 ± 0.2	
Lowest	11.6 ± 0.1	11.9 ± 0.1	10.4 ± 0.1		12.5 ± 0.1	9.7 ± 0.1		11.6 ± 0.1	11.6 ± 0.2	
**Allergic rhinitis (%)**	32.9 ± 0.1	29.1 ± 0.1	45.3 ± 0.2	< 0.001	-	-		30.5 ± 0.1	57.3 ± 0.3	< 0.001
**Asthma (%)**	9.0 ± 0.1	7.6 ± 0.0	13.5 ± 0.1	< 0.001	5.7 ± 0.0	15.6 ± 0.1	< 0.001	-	-	
**Atopic dermatitis (%)**	23.3 ± 0.1	-	-		19.0 ± 0.1	32.1 ± 0.1	< 0.001	22.1 ± 0.1	35.1 ± 0.2	< 0.001
**Oral symptoms (%)**	59.8 ± 0.1	58.3 ± 0.1	65.1 ± 0.2	< 0.001	57.6 ± 0.1	64.3 ± 0.1	< 0.001	59.4 ± 0.1	64.2 ± 0.2	< 0.001
**Daily tooth brushing (n)**	3.52 ± 0.0	3.53 ± 0.0	3.53 ± 0.0	0.035	3.52 ± 0.0	3.55 ± 0.0	< 0.001	3.53 ± 0.0	3.52 ± 0.0	0.019
**Teeth scaling (%)**	25.9 ± 0.1	25.2 ± 0.1	27.9 ± 0.2	< 0.001	24.2 ± 0.1	29.3 ± 0.2	< 0.001	25.6 ± 0.1	28.3 ± 0.3	< 0.001
**Soft drinks/soda intake (%)**				< 0.001			< 0.001			< 0.001
High consumers	8.5 ± 0.1	8.7 ± 0.1	7.8 ± 0.1		8.7 ± 0.1	8.1 ± 0.1		8.4 ± 0.1	9.4 ± 0.1	
Medium consumers	17.4 ± 0.1	17.6 ± 0.1	17.0 ± 0.1		17.6 ± 0.1	17.1 ± 0.1		17.4 ± 0.1	17.9 ± 0.2	
Low consumers	74.1 ± 0.1	73.7 ± 0.1	75.2 ± 0.2		73.8 ± 0.1	74.8 ± 0.2		74.2 ± 0.1	72.7 ± 0.2	
**Snack foods intake (%)**				0.007			0.154			< 0.001
High consumers	12.6 ± 0.1	12.6 ± 0.1	12.7 ± 0.1		12.5 ± 0.1	12.8 ± 0.1		12.6 ± 0.1	13.1 ± 0.2	
Medium consumers	27.3 ± 0.1	27.2 ± 0.1	27.7 ± 0.1		27.2 ± 0.1	27.5 ± 0.1		27.4 ± 0.1	26.4 ± 0.2	
Low consumers	60.0 ± 0.1	60.2 ± 0.1	59.6 ± 0.1		60.2 ± 0.1	59.7 ± 0.2		60.0 ± 0.1	60.6 ± 0.3	

Data are expressed as mean ± SD or % ± SE.

### Risk of having current AD according to the presence of oral symptoms

**Figure 1:**
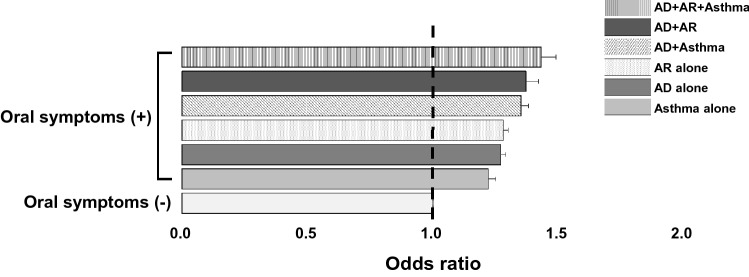
Risk for allergic diseases according to the presence of oral symptoms. When adjusted for confounders using model 2 (age, sex, region of residence, family income, smoking, stress, daily tooth brushing frequency, teeth scaling experience, soda/soft drink consumption, and snack foods consumption), the odds ratio (ORs) of current atopic dermatitis (AD) were significantly higher in those with oral symptoms than in those without such symptoms. The ORs increased with the combination of other allergic diseases, such as allergic rhinitis (AR) and/or asthma, compared to current AD alone. For all OR values, P < 0.001. A P-value less than 0.008 is significant after Bonferroni correction for multiple comparison. Abbreviations: AD, atopic dermatitis; AR, allergic rhinitis.

We examined the association between the presence of oral symptoms and the risk for current AD in Korean adolescents. Without adjusting for potential confounders, the ORs for current AD were significantly higher in those with oral symptoms (OR, 1.33; 95% confidence interval [CI], 1.32–1.35; *P* < 0.001) than in those without such symptoms. The ORs for current AD + AR, AD + AR + asthma, AR alone, and asthma alone were also significantly increased in participants with oral symptoms than in those without them (Supplementary Table 1). When adjusted for confounders using model 1 (age and sex) and model 2 (age, sex, region of residence, family income, and smoking, stress, daily tooth brushing frequency, teeth scaling experience, soft drinks/soda consumption, and snack foods consumption), the ORs remained significantly higher in participants with oral symptoms (Fig. 1). In model 2, the OR for current AD, AR, and asthma simultaneously (OR, 1.43; 95% CI, 1.36–1.50; P < 0.001) was higher than the ORs of current AD alone (OR, 1.27; 95% CI, 1.25–1.29; P < 0.001), AR alone (OR, 1.28; 95% CI, 1.26–1.30; P < 0.001), and asthma alone (OR, 1.22; 95% CI, 1.19–1.25; P < 0.001), respectively. Thus, the association between oral symptoms and current AD was significant using both adjustment models, and this relationship was also observed in AR, asthma, and their combination with AD.

In addition, we found that the prevalence of oral symptoms were higher in the 15–17 age group than in the 12–14 age group (62.5 ± 0.1% vs. 55.5 ± 0.1%, P < 0.001), and conversely, the OR for AD was higher in the 12–14 age group (OR, 1.30; 95% CI, 1.27–1.33, P < 0.001) than in the 15–17 age group (OR, 1.25; 95% CI, 1.22–1.28; P < 0.001). More detailed analysis is described in Supplementary Table 2.

### Differences in risk of having current AD, AR, and asthma according to specific oral symptoms

We also evaluated whether there were differences in the risk of having current AD, AR, and asthma according to each oral symptom (sensitive teeth, toothache, bleeding gums, and bad breath). All oral symptoms were significantly related with each of the allergic diseases (i.e., current AD, AR, and asthma) and their combination (Table 2). In model 2, the ORs for current AD with each oral symptom were higher in the following order: sensitive teeth (OR, 1.21; 95% CI, 1.19–1.22; *P* < 0.001), bad breath (OR, 1.18; 95% CI, 1.16–1.20; *P* < 0.001), toothache (OR, 1.16; 95% CI, 1.14–1.18; *P* < 0.001), and bleeding gums (OR, 1.14; 95% CI, 1.12–1.16; *P* < 0.001). In addition, the ORs for the combination of current AD, AR, and asthma in all oral symptoms were higher than those for current AD alone. Each oral symptom was significantly associated with the risk for current AD alone, AR alone, and asthma alone, and the ORs for the combination of these allergic diseases were higher than for any single allergic disease.

**Table 2:** Association between the presence of each oral symptom and allergic diseases in Korean adolescents.

Condition	Sensitive teeth		Toothaches		Bleeding gums, gum pain		Bad breath	
	OR (95% CI)	*P*-value	OR (95% CI)	*P-*value	OR (95% CI)	P-value	OR (95% CI)	*P*-value
**AD**								
Unadjusted	1.25 (1.24–1.27)	< 0.001	1.24 (1.23–1.26)	< 0.001	1.19 (1.17–1.21)	< 0.001	1.19 (1.18–1.21)	< 0.001
Model 1*	1.23 (1.21–1.25)	< 0.001	1.20 (1.18–1.22)	< 0.001	1.16 (1.14–1.18)	< 0.001	1.21 (1.19–1.23)	< 0.001
Model 2^†^	1.21 (1.19–1.22)	< 0.001	1.16 (1.14–1.18)	< 0.001	1.14 (1.12–1.16)	< 0.001	1.18 (1.16–1.20)	< 0.001
**AD + AR**								
Unadjusted	1.35 (1.33–1.38)	< 0.001	1.30 (1.28–1.33)	< 0.001	1.28 (1.26–1.31)	< 0.001	1.28 (1.25–1.30)	< 0.001
Model 1*	1.33 (1.30–1.35)	< 0.001	1.26 (1.24–1.29)	< 0.001	1.25 (1.23–1.28)	< 0.001	1.28 (1.25–1.31)	< 0.001
Model 2^†^	1.27 (1.24–1.30)	< 0.001	1.21 (1.18–1.24)	< 0.001	1.22 (1.19–1.26)	< 0.001	1.25 (1.22–1.28)	< 0.001
**AD + Asthma**								
Unadjusted	1.32 (1.28–1.37)	< 0.001	1.29 (1.24–1.34)	< 0.001	1.29 (1.24–1.34)	< 0.001	1.36 (1.31–1.40)	< 0.001
Model 1*	1.34 (1.30–1.38)	< 0.001	1.33 (1.28–1.38)	< 0.001	1.31 (1.27–1.36)	< 0.001	1.36 (1.31–1.41)	< 0.001
Model 2^†^	1.27 (1.22–1.32)	< 0.001	1.25 (1.20–1.30)	< 0.001	1.25 (1.20–1.31)	< 0.001	1.30 (1.25–1.36)	< 0.001
**AD + AR + Asthma**								
Unadjusted	1.42 (1.36–1.47)	< 0.001	1.36 (1.31–1.42)	< 0.001	1.37 (1.31–1.43)	< 0.001	1.41 (1.35–1.47)	< 0.001
Model 1*	1.43 (1.38–1.49)	< 0.001	1.39 (1.33–1.45)	< 0.001	1.39 (1.33–1.46)	< 0.001	1.41 (1.35–1.48)	< 0.001
Model 2^†^	1.34 (1.28–1.40)	< 0.001	1.30 (1.23–1.36)	< 0.001	1.33 (1.26–1.40)	< 0.001	1.33 (1.27–1.40)	< 0.001
**AR**								
Unadjusted	1.27 (1.25–1.30)	< 0.001	1.21 (1.20–1.23)	< 0.001	1.12 (1.18–1.22)	< 0.001	1.22 (1.20–1.23)	< 0.001
Model 1*	1.26 (1.24–1.28)	< 0.001	1.19 (1.18–1.21)	< 0.001	1.18 (1.17–1.20)	< 0.001	1.21 (1.19–1.23)	< 0.001
Model 2^†^	1.21 (1.19–1.23)	< 0.001	1.15 (1.13–1.17)	< 0.001	1.17 (1.15–1.18)	< 0.001	1.19 (1.17–1.21)	< 0.001
**Asthma**								
Unadjusted	1.20 (1.18–1.22)	< 0.001	1.17 (1.14–1.20)	< 0.001	1.19 (1.16–1.22)	< 0.001	1.23 (1.20–1.25)	< 0.001
Model 1*	1.23 (1.21–1.26)	< 0.001	1.23 (1.21–1.26)	< 0.001	1.23 (1.20–1.26)	< 0.001	1.23 (1.20–1.25)	< 0.001
Model 2^†^	1.18 (1.15–1.21)	< 0.001	1.18 (1.15–1.21)	< 0.001	1.19 (1.15–1.22)	< 0.001	1.18 (1.15–1.21)	< 0.001

*Model 1: adjusted for age and sex.

^†^Model 2: adjusted for age, sex, region of residence, family income, smoking, stress, daily tooth brushing frequency, teeth scaling experience, soft drinks/soda consumption, and snack foods consumption.

A *P*-value less than 0.008 is significant after Bonferroni correction for multiple comparison.

Abbreviations: AD, atopic dermatitis; AR, allergic rhinitis; OR, odds ratio; CI; confidence interval.

In age-subgroup analysis, all four oral symptoms were also associated with the presence of allergic diseases in both age group, and the ORs were also high in the order of sensitive teeth, bad breath, toothache, and bleeding gums in both age group. The ORs for allergic diseases were higher in the 12–14 age group than that in 15–17 age group (Supplementary Table 2).

## Discussion

**Figure 2:**
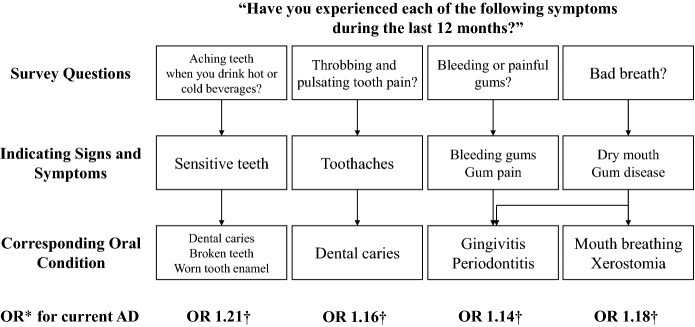
Schematic diagram of self-reported oral symptoms and each corresponding oral condition. We inferred the corresponding oral conditions from each survey question, and each OR value of current AD diagnosis was significantly higher for all four oral symptoms. ^*^Adjusted for age, sex, region of residence, family income, smoking, stress, daily tooth brushing frequency, teeth scaling experience, soda/soft drink consumption, and snack foods consumption. ^†^P < 0.001. Abbreviations: AD, atopic dermatitis; OR, odds ratio.

In this study, we found that the presence of oral symptoms was associated with increased risk for physician-diagnosed current AD in Korean adolescents aged 12–18 years. The oral symptoms in the questionnaire included sensitive teeth, toothache, bleeding gums, and bad breath, which may indicate broken teeth or worn tooth enamel, dental caries, gingivitis or periodontitis, and dry mouth or gum disease, respectively. When analyzed for each oral symptom, all four oral symptoms were also significantly associated with current AD (Fig. 2). A further analysis revealed the ORs for having two or more allergic diseases, such as AD + AR, AD + asthma, and AD + AR + asthma, were higher than that of AD alone in participants with oral symptoms. When compared 12–14 age group and 15–17 age group, the ORs for AD and/or other allergic diseases were higher in the 12–14 age group, which showed a lower prevalence of oral symptoms than 15–17 age group. There was no significant difference in the prevalence of AD between the age subgroup, so the cause of this age-related difference is uncertain.

Previous studies regarding oral manifestations in AD, most of which have been cross-sectional studies, have suggested that the following oral manifestations may be associated with AD: increased susceptibility to cariogenic activity, odontogenic focal infection, and reactivation of herpes simplex virus (HSV) type 1 followed by release into the oral cavity^16–19^. According to a study based on a national survey that enrolled 91,642 children aged 0–17 years in the United States, eczema was associated with toothache (*P* < 0.0001), broken teeth (*P* = 0.01) and bleeding gums (*P* < 0.0001) but not with dental caries^20^. This study differed from our study in the questionnaire items, directly including the presence of dental caries and broken teeth.

A study of 21,792 Japanese children aged 6–15 years indicated no significant associations between dental caries and allergic diseases, such as AD, AR, and asthma^21^. In this study, dental caries were defined as having one or more decayed or filled teeth on oral examination^21^. The different results from our study may arise from objective oral findings which is more strict outcomes than subjective oral symptoms. However, the GUSTO birth cohort study, which also included oral examination, found that a risk of early childhood caries (ECC), a chronic diet-mediated infectious oral disease at 2 or 3 years of age, was associated with the development of AD within the first year of birth^22^. This birth cohort study suggests the presence of a common pathologic mechanisms between AD and ECC, which is ectodermal defects during tissue development^22^. AD with positive skin prick test (SPT) results showed higher OR value than total reported-AD or AD with negative SPT results, which may exclude simple rashes or other skin condition other than true AD by combining SPT results^22^. Since our study investigated self-reported diseases only, there is a possibility that skin conditions other than AD may be included, resulting lower OR values of AD than GUSTO study which present OR value up to 3 in AD with positive SPT.

In addition, according to a study using National Health Insurance Database of Taiwan, the prevalence of AR, but not asthma, was associated with oral diseases such as dental caries, periodontitis, and gingivitis in the early 20s^23^. Similarly, Taiwanese children aged 1–9 years showed positive association between the frequency of clinical visit for dental caries and AR, but not asthma^24^. In Taiwanese studies, AR and asthma were analyzed by including each other in the adjustment model, while our study performed analysis by categorizing as AR, AR + asthma, etc. Although previous studies and our study have showed some inconsistent results, oral symptoms and AD seem to be associated in children and adolescents. The inconsistencies may be due to difference in study design, enrolled population (age and race), and definition and determination of each dental condition (different questionnaire or the presence of the oral examination).

Barrier dysfunction may contribute to increased susceptibility to microbial infection of both skin and the oral cavity. In this regard, there are several possible explanations for the relationship between AD and oral symptoms. AD patients are susceptible to cutaneous infection and colonization, particularly by *Staphylococcus aureus* and HSV^25^. Such vulnerability of AD patients to infection is based on skin barrier dysfunction caused by defects in structural proteins such as FLG, and immune dysregulation, including decreased levels of antimicrobial peptides, which may also result in susceptibility to oral cavity infection^1,25^. Similarly, the pathomechanism of ECC also includes some structural defects in tooth formative stages caused by genetic mutation, leading to increased susceptibility to dental caries^26^. For example, the distal-less homeobox (Dlx-3) gene is important for both enamel formation and epidermal differentiation, and some polymorphisms of genes, such as mannose-binding lectin 2 (MBL2) and toll-like receptor 2 (TLR2), are associated with both ECC and AD^22,27^. In addition, FLG, a key structural protein involved in barrier function, is expressed not only in the epidermis but also in the human oral mucosa^15^, which may cause more infections in the oral cavity. Therefore, increased susceptibility to infections caused by structural gene defects may be one of the mechanisms underlying the association between oral symptoms such as dental caries and AD.

Periodontitis is also an infectious disease, mainly caused by *Porphyromonas gingivalis*, *Treponema denticola*, and *Actinobacillus actinomycetemcomitans*^28,29^. In the human body, the second largest number of microbiota lives in the oral cavity, and when oral microbiota dysbiosis occurs, proliferation of the above-described anaerobic gram-negative bacteria trigger the immune response of the host, causing chronic inflammation and periodontal tissue destruction^30,31^. Periodontal diseases include gingivitis, which is more common in children, and periodontitis, which is more prevalent in adults^28^. Bleeding gums or gum pain may indicate periodontal disease, and are main signs of gingivitis^28^. Our findings suggest that gingivitis may be associated with increased risk for current AD, and this may also be explained by susceptibility to infection due to structural defects. When pathogens are propagated in the gingival crevice, proinflammatory cytokines and matrix metalloproteinases are released, and then local inflammation and periodontal destruction occur^28^. Microbial antigens and proinflammatory cytokines could enter the systemic circulation and cause systemic inflammation as well as local inflammation^32^. This suggests the possibility of oral lesions as an aggravating factor for AD. Unfortunately, we could not determine the association between oral symptoms and the severity of AD due to the limitations of the questionnaire. However, a previous study indicated the improvement of symptoms in AD patients treated for odontogenic focal infections for 3 months, even when there were no complaints regarding oral symptoms^18^. Further studies are required to clarify the association between oral manifestations and AD severity, and the disease course of AD after treatment of oral lesions.

Our study also showed that self-reported bad breath was associated with current AD. Bad breath may be a sign of not only gum disease but also dry mouth, which is due to mouth breathing or xerostomia^29^. Although, a decreased salivary flow was observed in asthma and AR patients, it is unclear whether this finding is related to the pathological mechanism of the disease itself, or a result of the treatment of the disease, because some medications, such as antihistamines and inhalers, can cause xerostomia^33,34^. There are no studies that have directly investigated salivary flow in AD patients, but there has been a study that demonstrated significantly increased OR for mouth breathing in AD patients aged 2–6 years even after adjusting for the coexistence of AR or asthma^35^. Decreased saliva resulting from mouth breathing or antihistamines could increase susceptibility to oral infection because saliva plays an important role in host defense against oral pathogens^36^. In addition, *Candida albicans*, which is a commensal yeast normally present in the oral cavity, may be transformed to a pathogenic form, resulting in the developing of oral lesions, because AD patients are often treated with immunosuppressants or antibiotics and may also have xerostomia^19,37^. A decrease in IgA levels has been also found in gingival tissue of asthma patients, which plays an important role in immune defense at mucosal levels, thus this may be involved with susceptibility to oral infection in allergic patients^38^. Furthermore, as observed in the FLG-defective skin, oral mucosa with defective FLG may also be susceptible to dryness and infection leading to dental caries^19,39^.

This study had some limitations. First, it was based on the results of a self-reported survey and no physical examinations or objective tests were performed. Second, although we found significant associations between patient-reported oral symptoms and current AD, our investigation could not reveal causal links due to the limitations of the study design. Since nebulizers, inhalers and oral medications such as antihistamines may affect xerostomia or other oral conditions, it is also a limitation that the analysis conducted without medication history. Additionally, the relationships between oral symptoms and severity of AD or aggravation of AD could not be established. Finally, because of its large samples size and small effect size, there is also a risk of overpowered study which may be vulnerable to an inflated false positive rate.

In summary, we found that oral symptoms, such as sensitive teeth, toothache, bleeding gums or gum pain, and bad breath, were associated with increased risk for current AD. Although the magnitudes of OR in our study seem to be not strong, it is still significant, and we believe it suggests substantial association between oral symptoms and AD. This study alone is insufficient to determine whether altered oral condition and AD are sharing a common pathogenic pathway, or whether these findings are more likely results or complications of AD, and other allergic diseases. We provide plausible explanations as follows: sensitive teeth, toothache, bleeding gums, and bad breath may be caused by structural defects of enamel and oral epidermis/mucosa resulting increased susceptibility to oral infections, and bad breath may be the results of decreased salivary flow due to mouth breathing or antihistamines, and toothache, bleeding gums, and bad breath may also be induced by change in normal microbiota by the use of immunosuppressants in AD patients. These oral symptoms seem to be interconnected. We therefore suggest that children and adolescents with AD should undergo regular dental examinations and receive appropriate treatment as necessary.

## Methods

### Study population

Since 2005, the Korea Centers for Disease Control and Prevention (CDC) has annually conducted a large-scale web-based survey to monitor health risk behaviors of Korean middle and high school students, known as the Korea Youth Risk Behavior Web-based Survey (KYRBS)^40^. KYRBS adopts a stratified, clustered, multi-stage (geographic area, school size, and grade) probability sampling design and the response rate has been high as over 95%^40^. This survey is therefore considered a nationally representative sample of general Korean adolescents aged 12–18 years^40,41^. The KYRBS was approved by the Institutional Review Board of the Korea Centers for Disease Control and Prevention (Statistics Korea, approval No.11758). All participants signed online informed consent forms^40^. All methods in the study were carried out with relevant guidelines and regulations.

We analyzed the data from nine survey years (KYRBS 2009–2017) including 638,475 individuals. Among them, we excluded the participants who did not answer any of the following: region of residence, family income, smoking, stress, school performance, oral symptoms, and the physician-diagnosed AD, AR, and asthma. Finally, a total of 634,299 participants was enrolled in this study.

### Variable definitions

The following question was used for each participant to determine current AD, AR, and asthma: “Have you been diagnosed with AD (or AR or asthma) by a doctor within the last 12 months?” Oral symptoms were assessed using the question “Have you experienced any of the following symptoms during the last 12 months?”: aching teeth when you drink hot or cold beverages; throbbing and pulsating tooth pain; bleeding or painful gums; or bad breath. We considered participants with any of the above four symptoms as having an oral symptom. The region of residence was classified as large city, medium or small city, and rural area. For family income, level of stress, and school performance, each participant made a self-assessment by responding to questions “How would you describe your academic performance (or family income/level of stress)?” into one of the following five levels: highest, upper-middle, middle, lower-middle, and lowest. Smoking status was classified as current cigarette smoking or not. The number of tooth brushing per day and tooth scaling within the last year were also evaluated. According to the weekly frequency of consumption of soft drinks/soda or snack foods, the participants were classified as low (0–2 times/week), medium (3–4 times/week) or high (more than 5 times/week) soft drinks/soda or snack foods consumers, respectively.

Although validity tests have not been performed for all variables, Korea CDC conducted a validity test for the self-reported height, weight and smoking status, and the results showed a good validity.

### Statistical analyses

Sampling weights, stratification, and clusters provided in the KYRBS data set were incorporated into the analysis, to account for the complex KYRBS survey design and obtain proper estimates and their standard errors. The differences in general characteristics of the participants according to the presence of each atopic disease were analyzed with a *t*-test using the complex samples general linear model for continuous variables and Pearson χ^2^ test with Rao-Scott adjustment for categorical variables. To estimate ORs for each of AD, AR, and asthma and their combinations (AD + AR, AD + asthma, and AD + AR + asthma) according to the presence of oral symptoms, we performed binary logistic regression (univariate and multivariate analyses) with a complex sampling design in the following ways: no adjustment, confounder adjustment for age and sex (model 1), and for age, sex, region of residence, family income, smoking, stress, daily tooth brushing frequency, teeth scaling experience, soda/soft drink consumption, and snack foods consumption (model 2). Potential co-variates for model 2 were adopted based on previous studies on the epidemiology and risk of allergic diseases using large-scale nationwide population-based data^42–44^, and then final co-variates were selected via univariate analyses which determined each variable’s significance. Multicollinearity among independent variables also checked by the calculation of the variance inflation factor (VIF)^45^.

For subgroup analyses according to the presence of each oral symptom, ORs for AD alone, AR alone, asthma alone, and their combinations were also obtained by similar logistic regression analyses with complex sampling. Values are expressed as means ± standard deviations (SD) or proportions ± standard errors (SE), and *P* value < 0.05 was taken to indicate significance. For multiple comparison, we determined proper *P* value via the Bonferroni correction. All statistical analyses were performed using SPSS version 22.0 (IBM, Armonk, NY).

## Supplementary information

Supplementary Information.

## Data Availability

The datasets generated during and/or analyzed during the current study are available from the corresponding author on reasonable request.
