# Supplementary material for: Identification of oral symptoms associated with atopic dermatitis in adolescents: Results from the Korea national representative survey 2009–2017
Source: Sci Rep. 2020 Nov 10;10:19461. doi: 10.1038/s41598-020-76532-1 (PMC7655854; doi:10.1038/s41598-020-76532-1)
Supplement: Supplementary file 1 — Supplementary Information. [file 41598_2020_76532_MOESM1_ESM.docx]

**[Title page for supplementary files]**

**Identification of oral symptoms associated with atopic dermatitis in adolescents: Results from the Korea national representative survey 2009-2017**

Ji-Su Shim^1^ and Min-Suk Yang^2,3*^

^1^Division of Allergy and Clinical Immunology, Department of Internal Medicine, Ewha Womans University College of Medicine, Seoul, Korea

^2^Division of Allergy and Clinical Immunology, Department of Internal Medicine, Seoul National University College of Medicine, Seoul, Korea

^3^Department of Internal Medicine, SMG-SNU Boramae Medical Center, Seoul, Korea

**Running title:** Impaired oral health in atopic dermatitis

***Corresponding author:*** Min-Suk Yang, MD, PhD

Department of Internal medicine, SMG-SNU Boramae Medical Center

5 Gil 20 Boramae-road, Dongjak-gu, Seoul, Korea, 07061

Tel: 82-2-870-2237, Fax: 82-2-831-0714

E-mail: iatrus13@hanmail.net

**Supplementary Table 1. Association between the presence of oral symptoms and allergic diseases in Korean adolescents.**

| Condition | Unadjusted | | Model 1^*^ | | Model 2^†^ | |
| --- | --- | --- | --- | --- | --- | --- |
|  | **OR (95% CI)** | ***P*-value** | **aOR**^*^ **(95% CI)** | ***P*-value** | **aOR**^†^ **(95% CI)** | ***P*-value** |
| AD | 1.33 (1.32-1.35) | <0.001 | 1.30 (1.28-1.32) | <0.001 | 1.27 (1.25-1.29) | <0.001 |
| AD + AR | 1.44 (1.41-1.47) | <0.001 | 1.41 (1.38-1.44) | <0.001 | 1.37 (1.34-1.40) | <0.001 |
| AD + Asthma | 1.38 (1.33-1.43) | <0.001 | 1.41 (1.36-1.46) | <0.001 | 1.35 (1.30-1.41) | <0.001 |
| AD + AR + Asthma | 1.48 (1.42-1.54) | <0.001 | 1.50 (1.44-1.60) | <0.001 | 1.43 (1.36-1.50) | <0.001 |
| AR | 1.33 (1.31-1.34) | <0.001 | 1.31 (1.30-1.33) | <0.001 | 1.28 (1.26-1.30) | <0.001 |
| Asthma | 1.23 (1.20-1.25) | <0.001 | 1.27 (1.25-1.30) | <0.001 | 1.22 (1.19-1.25) | <0.001 |

^*^Model 1: adjusted for age and sex.

^†^Model 2: adjusted for age, sex, region of residence, family income, smoking, stress, daily tooth brushing frequency, teeth scaling experience, soft drinks/soda consumption, and snack foods consumption.

A *P*-value less than 0.008 is significant after Bonferroni correction for multiple comparison.

Abbreviations: AD, atopic dermatitis; AR, allergic rhinitis; OR, odds ratio; CI; confidence interval.

**Supplementary Table 2. Age-Subgroup analysis: association between the presence of oral symptom and allergic diseases in Korean adolescents**

| **Condition** | | **Total oral symptoms** | | **Each oral symptoms** | | | | | | | |
| --- | --- | --- | --- | --- | --- | --- | --- | --- | --- | --- | --- |
|  |  |  |  | **Sensitive teeth** | | **Toothaches** | | **Bleeding gums, gum pain** | | **Bad breath** | |
|  |  | **OR (95% CI)** | ***P*-value** | **OR (95% CI)** | ***P*-value** | **OR (95% CI)** | ***P-*value** | **OR (95% CI)** | **P-value** | **OR (95% CI)** | ***P*-value** |
| **AD** | |  | |  | |  | |  | |  | |
|  | Aged 12-14 | 1.30 (1.27-1.33) | <0.001 | 1.23 (1.20-1.26) | <0.001 | 1.20 (1.16-1.23) | <0.001 | 1.17 (1.14-1.21) | <0.001 | 1.22 (1.18-1.25) | <0.001 |
|  | Aged 15-17 | 1.25 (1.22-1.28) | <0.001 | 1.18 (1.16-1.21) | <0.001 | 1.14 (1.11-1.17) | <0.001 | 1.13 (1.10-1.16) | <0.001 | 1.16 (1.13-1.19) | <0.001 |
| **AD + AR** | |  |  |  |  |  |  |  |  |  |  |
|  | Aged 12-14 | 1.40 (1.35-1.46) | <0.001 | 1.32 (1.27-1.36) | <0.001 | 1.27 (1.22-1.32) | <0.001 | 1.24 (1.19-1.29) | <0.001 | 1.29 (1.24-1.34) | <0.001 |
|  | Aged 15-17 | 1.34 (1.29-1.38) | <0.001 | 1.23 (1.20-1.27) | <0.001 | 1.18 (1.14-1.22) | <0.001 | 1.22 (1.18-1.26) | <0.001 | 1.22 (1.18-1.27) | <0.001 |
| **AD + asthma** | |  |  |  |  |  |  |  |  |  |  |
|  | Aged 12-14 | 1.40 (1.32-1.49) | <0.001 | 1.34 (1.27-1.41) | <0.001 | 1.27 (1.19-1.35) | <0.001 | 1.22 (1.14-1.31) | <0.001 | 1.35 (1.28-1.44) | <0.001 |
|  | Aged 15-17 | 1.29 (1.22-1.37) | <0.001 | 1.20 (1.14-1.27) | <0.001 | 1.24 (1.16-1.31) | <0.001 | 1.28 (1.21-1.36) | <0.001 | 1.25 (1.18-1.33) | <0.001 |
| **AD + AR + asthma** | |  |  |  |  |  |  |  |  |  |  |
|  | Aged 12-14 | 1.46 (1.36-1.58) | <0.001 | 1.42 (1.33-1.52) | <0.001 | 1.33 (1.23-1.44) | <0.001 | 1.29 (1.19-1.40) | <0.001 | 1.38 (1.28-1.49) | <0.001 |
|  | Aged 15-17 | 1.39 (1.29-1.46) | <0.001 | 1.25 (1.17-1.33) | <0.001 | 1.27 (1.18-1.36) | <0.001 | 1.36 (1.26-1.46) | <0.001 | 1.28 (1.19-1.38) | <0.001 |
| **AR** | |  |  |  |  |  |  |  |  |  |  |
|  | Aged 12-14 | 1.33 (1.29-1.36) | <0.001 | 1.26 (1.23-1.29) | <0.001 | 1.20 (1.17-1.24) | <0.001 | 1.19 (1.16-1.23) | <0.001 | 1.21 (1.18-1.25) | <0.001 |
|  | Aged 15-17 | 1.25 (1.22-1.27) | <0.001 | 1.17 (1.16-1.20) | <0.001 | 1.13 (1.11-1.15) | <0.001 | 1.16 (1.13-1.18) | <0.001 | 1.18 (1.15-1.21) | <0.001 |
| **Asthma** | |  |  |  |  |  |  |  |  |  |  |
|  | Aged 12-14 | 1.28 (1.23-1.32) | <0.001 | 1.24 (1.19-1.28) | <0.001 | 1.22 (1.17-1.26) | <0.001 | 1.21 (1.16-1.26) | <0.001 | 1.22 (1.17-1.27) | <0.001 |
|  | Aged 15-17 | 1.18 (1.14-1.22) | <0.001 | 1.13 (1.09-1.17) | <0.001 | 1.16 (1.12-1.20) | <0.001 | 1.16 (1.13-1.22) | <0.001 | 1.15 (1.11-1.20) | <0.001 |

All models were adjusted for sex, region of residence, family income, smoking, stress, daily tooth brushing frequency, teeth scaling experience, soft drinks/soda consumption, and snack foods consumption.

A P-value less than 0.008 is significant after Bonferroni correction for multiple comparison.

Abbreviations: AD, atopic dermatitis; AR, allergic rhinitis; OR, odds ratio; CI; confidence interval
